# Effects of propofol on intracranial pressure and prognosis in patients with severe brain diseases undergoing endotracheal suctioning

**DOI:** 10.1186/s12883-020-01972-1

**Published:** 2020-10-29

**Authors:** Menghang Wu, Xiaorong Yin, Maojun Chen, Yan Liu, Xia Zhang, Tingting Li, Yujuan Long, Xiaomei Wu, Lihui Pu, Maojie Zhang, Zhi Hu, Ling Ye

**Affiliations:** 1 West China Hospital, Sichuan University/West China School of Nursing, Sichuan University, Chengdu, Sichuan Province 610041 P. R. China; 2 Department of Pain Management, West China Hospital, Sichuan University, Chengdu, Sichuan Province 610041 P. R. China

**Keywords:** Severe neuropathy, Severe brain disease, Endotracheal suctioning (ES), Propofol, Sedation, Intracranial pressure (ICP), Nursing

## Abstract

**Background:**

To investigate whether the administration of intravenous propofol before endotracheal suctioning (ES) in patients with severe brain disease can reduce the sputum suction response, improve prognosis, and accelerate recovery.

**Methods:**

A total of 208 severe brain disease patients after craniocerebral surgery were enrolled in the study. The subjects were randomly assigned to the experimental group (*n* = 104) and the control group (*n* = 104). The experimental group was given intravenous propofol (10 ml propofol with 1 ml 2% lidocaine), 0.5–1 mg/kg, before ES, while the control group was subjected to ES only. Changes in vital signs, sputum suction effect, the fluctuation range of intracranial pressure (ICP) before and after ES, choking cough response, short-term complications, length of stay, and hospitalization cost were evaluated. Additionally, the Glasgow Outcome Scale (GOS) prognosis score was obtained at 6 months after the operation.

**Results:**

At the baseline, the characteristics of the two groups were comparable (*P* > 0.05). The increase of systolic blood pressure after ES was higher in the control group than in the experimental group (*P* < 0.05). The average peak value of ICP in the experimental group during the suctioning (15.57 ± 12.31 mmHg) was lower than in the control group (18.24 ± 8.99 mmHg; *P* < 0.05). The percentage of patients experiencing cough reaction- during suctioning in the experimental group was lower than in the control group (*P* < 0.05), and the fluctuation range of ICP was increased (*P* < 0.0001). The effect of ES was achieved in both groups. The incidence of short-term complications in the two groups was comparable (*P* > 0.05). At 6 months after the surgery, the GOS scores were significantly higher in the experimental than in the control group (4–5 points, 51.54% vs. 32.64%; 1–3 points, 48.46% vs. 67.36%; *P* < 0.05). There was no significant difference in the length of stay and hospitalization cost between the two groups.

**Conclusions:**

Propofol sedation before ES could reduce choking cough response and intracranial hypertension response. The use of propofol was safe and improved the long-term prognosis.

The study was registered in the Chinese Clinical Trial Registry on May 16, 2015 (ChiCTR-IOR-15006441).

## Background

Severe brain disease is often accompanied by disorders of consciousness, weak sputum discharge by spontaneous cough, airway obstruction, and hypoxia, which together aggravate secondary damage to brain cells [1–3]. To maintain airway patency and avoid airway obstruction and pulmonary infection in patients affected by the severe brain disease, artificial airways should be established, and endotracheal suctioning (ES) should be timely repeatedly performed [1, 4, 5].

ES stimulates airway mucosa, triggers cough reflex, induces bronchospasm, decreases blood oxygen saturation, and increases intracranial pressure. However, severe airway stimulation may lead to adverse consequences [1], such as severe cough, increased chest pressure, a sudden rise in blood pressure, increased cerebral perfusion, increased intracranial pressure (ICP) caused by cerebral vasospasm, and increased risk of vascular rupture [6–8]. The stimulation of the airway caused by different suction modes and duration, the amount of negative pressure applied, and the depth of suction tube insertion lead to reflexive ICP changes [4–6, 9–12].

Propofol, a short-term acting sedative, can reduce the cerebral blood flow, ICP, and cerebral metabolic rate of oxygen (CMRO2). The action of propofol is characterized by a fast onset time of approximately 30–60 s, a short half-life of 10–15 min, and a fast wake-up time after drug withdrawal, which facilitates the evaluation of the nervous system [13–16]. During the ES process, propofol can directly dilate the bronchial smooth muscles, inhibit the pharyngeal reflex, and reduce the airway hyperresponsiveness [13, 14, 16]. In addition, it exerts amnestic and anticonvulsant effects, increasing the comfort of patients [17, 18]. Moderate or slow infusion (respectively, 40 mg/10s or 20–50 mg/min in generally healthy adults) has no significant effect on the vital signs of patients [13, 14, 17–19].

The objective of the present study was to explore whether the administration of propofol before the ES procedure in severe brain disease patients would help to maintain the respiratory and circulatory stability, reduce the increase of ICP, and suppress the high-pressure response caused by the intense stimulation.

## Methods

### Study participants

This study has been approved by the Clinical Trial and Biomedical Ethics Committee of the West China Hospital of Sichuan University (approval number 2014 (238)). All patients signed informed consent. A total of 208 severe brain disease patients who underwent craniocerebral surgery in the West China Hospital of Sichuan University from May 2015 to October 2018 were included (clinical trial registration number: ChiCTR-IOR-15006441). Patients were assigned to the experimental group and the control group according to the random number generated by the computerized random number table. The inclusion criteria were: patients (1) aged 18–75 years; (2) with cerebrovascular disease and undergoing craniocerebral surgery, including cerebrovascular diseases, intracranial tumors and severe brain injury, according to the diagnostic criteria of severe brain disease [20]; (3) with artificial airway and ventilator-assisted respiration; (4) equipped with intracranial pressure monitor; and (5) with the initial ICP of ≤25 mmHg. The exclusion criteria included: patients with (1) insufficient blood volume or unstable circulation; (2) hypotension; (3) shock; or (4) maternal patients.

### Research methods

Severe brain disease in this study included cerebrovascular disease, intracranial tumors, severe brain injury et al. And the patients were divided into various degrees according to the Glasgow Outcome Scale (GOS). Patients in the both groups were treated by the same team of doctors and nurses. After the operation, both groups were treated with anti-inflammatory medications, ICP-reducing drugs, and nutritional support. All patients were subjected to the continuous ECG monitoring, oxygen inhalation. And the intracranial pressure monitor was installed beside the bed, so the digital changes and fluctuations of intracranial pressure could be observed directly. The control group was given ES directly without prior administration of propofol. The experimental group was sedated with propofol before ES. The dose of propofol was 0.5–1 mg/kg (10 ml propofol with 1 ml 2% lidocaine), and the injection was performed slowly. The patients were under sedation condition. And the doctors in our team were anesthesiologist qualified to manage the person at whatever level of sedation or anesthesia. For the ES procedure, patients were placed in a supine position, and the head of the bed was raised 15–30°. During the operation, No. 12 sputum suction tubes were used, the interval between consecutive ES was more than 30 min, and the negative pressure was set to 200 mmHg; the deep ES was performed [2, 3, 21]. Each patient had ES applied at least 5 times.

### Outcome measures

The changes in the vital signs, ES effect, the fluctuation range of ICP before and after ES, choking and coughing reaction, recent complications, prognosis score measured by the GOS 6 months after the procedure, the duration of in-hospital stay, and hospitalization expenses were compared between the two groups.

#### ES indications ES

Was considered necessary in the following cases: rapid breathing, high blood pressure, high airway pressure, cough, decreased SPO_2_, presence of secretions in the airway, and wheezy phlegm on auscultation [2, 3, 22].

#### Vital signs and SPO_2_

The vital signs and SPO_2_ were determined within 5 min after ES [17, 18].

#### Measurement of ICP (mm H_2_O) fluctuation ranges

The ICP fluctuation range was evaluated by the peak value of ICP during ES, the time to reach the peak value (seconds), the value of ICP after the recovery to a stable state, and the time to recover to a stable state (seconds).

#### Assessment of choking cough response

The choking cough response was graded as follows: grade 1, no choking cough; grade 2, slight cough, 1–2 times, without apparent physical movement; grade 3, strong cough, 3–4 times, with neck and chest movement; grade 4, more than four coughs, accompanied by movement of the entire body and retching, and causing extreme pain [23].

#### Auscultation evaluation of the ES effect

Three degrees of reduction of the wheeze phlegm were assigned: 1, complete disappearance; 2, significant decrease; and 3, partial decrease.

#### GOS

Six months after the procedure, the patients were evaluated using the GOS prognosis score [23, 24]. The GOS scores of 4 and 5 indicated a good prognosis, and scores of 1–3 indicated poor prognosis [23–27].

### Statistical analysis

The baseline measurement data were analyzed using the SPSS 22.0 software and were represented as the mean and standard deviation (*x ± s*). The Student’s t-test was used for comparisons between the two groups. The enumeration data were represented as the composition ratio or percentage, and the chi-square test or Fisher’s exact probability method was used for inter-group comparison. The rank data comparison was performed using the rank-sum test. The significant level was set at α = 0.05 (two-tailed), and *P* < 0.05 was considered statistically significant. Repeated measurement of quantitative data was analyzed by the SAS software. A random intercept-slope model that included grouping variables and measurement times was established.

## Results

### Comparison of baseline conditions between the two groups

A total of 206 patients were included (2 patients in the control group withdrew from the study). The average age of the 104 patients in the experimental group was 52.45 ± 15.05 years, and the average age of the 102 patients in the control group was 52.68 ± 14.06 years. There was no significant difference in the age, gender, condition (pupil size, consciousness, tracheal situation), disease classification, surgical method, and GOS between the two groups (all *P* > 0.05) (Table 1).

**Table 1:** Comparison of general conditions between the two groups

Clinical data	Experimental group (*n* = 104)		Control group (*n* = 102)		*P*
Gender					
Male	48		55		0.2649
Female	56		47		
Age	52.45 ± 15.05		52.68 ± 14.06		0.9120
Weight	60.82 ± 11.26		64.24 ± 11.31		0.0315
Pupil					
Diameter	Left: 2.3204	Right: 2.4412	Left: 2.3235	Right: 2.4412	0.9872
Light reflection					0.6264
Consciousness					
Sober	3		5		0.3273
Drowsiness	16		15		
Lethargy	28		21		
Light coma	29		30		
Coma	27		31		
Deep coma	0		0		
Trachea condition					
Endotracheal intubation	104		102		0.4976
Tracheotomy	34/104 (33.01%)		32/102 (31.37%)		0.8019
Disease classification / cases (%)					
Cerebrovascular diseases	74 (71.15%)		68 (66.67%)		0.3914
Intracranial tumors	22 (21.15%)		19 (18.63%)		
Severe brain injury	7 (6.73%)		14 (13.73%)		
Other	1 (0.96%)		1 (0.98%)		
Surgical method / cases (%)					
Decompressive osteotomy	2 (1.93%)		4 (6.86%)		0.1237
Hematoma removal + decompressive osteotomy	28 (25.96%)		42 (35.29%)		
Aneurysm clipping or vascular malformation resection	49 (47.12%)		39 (38.24%)		
Tumor resection	25 (25.00%)		17 (16.67%)		
APACH score					0.9679

### Effect of propofol on vital signs

Before the administration of propofol and ES, the vital signs were comparable between the two groups (*P* > 0.05). After ES, the systolic pressure in the control group was higher than in the experimental group (*P* < 0.05), while the values of HR, P, SpO2, and diastolic pressure were similar in both groups (all *P* > 0.05 (Table 2).

**Table 2:** Comparison of vital signs between the two groups before and after ES

Before						After				
	HR	P	SpO2	Systolic pressure (mmHg)	Diastolic pressure (mmHg)	HR	P	SpO2	Systolic pressure (mmHg)	Diastolic pressure (mmHg)
Experimental group	78.75	14	100	134.71	72.56	89.5	21	100	139.24	75.85
Control group	77.75	15	100	136.44	71.53	93.5	24	100	144.93	76.30
t	−0.32	1.75	0.15	−0.51	−1.89	0.68	0.9	1.43	2.68	0.49
P	0.75	0.081	0.88	0.61	0.06	0.49	0.37	0.15	0.008	0.62

### Effect of propofol on ICP

Before and after the ES, the differences in ICP between the two groups were not significant (*P* > 0.05). The average peak value of ICP during ES in the experimental group (15.57 ± 12.31 mmHg) was lower than in the control group (18.24 ± 8.99 mmHg, *P* < 0.05) (Table 3).

**Table 3:** Comparison of ICP fluctuation between the two groups

Group	ICP before ES (mmHg)	ICP during ES (mmHg)	ICP after ES (mmHg)
Experimental group	8.88 ± 8.57	15.57 ± 12.31	8.91 ± 8.70
Control group	8.68 ± 8.23	18.24 ± 8.99	9.00 ± 8.53
*t*	0.19	4.80	1.86
*P*	0.848	< 0.0001	0.065

### Effect of propofol on choking cough response and ICP fluctuation

The beneficial effect of ES was observed in both groups of patients (P > 0.05) (Table 4). However, the proportion of patients suffering from pain in the experimental group was lower than in the control group (grade 3: 27.39% vs. 36.72%; grade 4: 0.12% vs. 2.15%. The grade of choking cough reaction was directly related to the fluctuation range of ICP (*P* < 0.0001) (Table 4).

**Table 4:** Comparison of ES effect and choking cough reaction between the two groups (case)

Group	ES effect, n (%)			Choking cough response, n (%)				ICP fluctuation range (mmHg)
	1	2	3	1	2	3	4	
Experimental group	8 (0.93%)	625 (72.76%)	226 (26.34%)	82 (9.56%)	540 (62.94%)	235 (27.39%)	1 (0.12%)	6.68 ± 7.02
Control group	1 (0.11%)	656 (74.12%)	228 (25.76%)	34 (3.84%)	507 (57.29%)	325 (36.72%)	19 (2.15%)	9.56 ± 5.09
P	0.99			< 0.01				< 0.0001

### Effect of propofol of complications and prognosis

#### Comparison of the incidence of complications between the two groups

The number of the complications in the two groups is listed in Table 5. There were no significant differences in the number of cases of cerebral hemorrhage, brain hernia, and pulmonary infection (all *P* > 0.05).

**Table 5:** Comparison of complications between the two groups (% (n)/ X ± s)

Group	Cerebral hemorrhage	Brain hernia	Pulmonary infection
Experimental group	0	3	38
Control group	4	10	48
Statistical quantity	104	101	2.34
P	1.00	0.99	0.12

Note: patients can have two or more complications at the same time

#### Comparison of GOS scores between two groups

Six months after the procedure, 51.54% of the patients in the experimental group and 32.64% in the control group had the GOS score of 4 or 5, while 48.46% in the experimental group and 67.36% in the control group had the GOS score of 1–3. The cases of 4–5 and 1–3 points in the experimental group were both significantly less than the control group (both *P* < 0.05) (Table 6).

**Table 6:** Comparison of GOS scores between the two groups, *n* (%)

Group	1 point	2 points	3 points	4 points	5 points	*P*
Experimental group	8 (8.25%)	7 (7.22%)	32 (32.99%)	21 (21.65%)	29 (29.90%)	0.037
Control group	17 (17.89%)	5 (5.26%)	42 (44.21%)	18 (18.95%)	13 (13.68%)	

### Effect of propofol on hospital length of stay and cost

There was no statistically significant difference between the two groups in total hospital expenses and the length of in-hospital stay (both *P* > 0.05).

## Discussion

The results of the present investigation documented that propofol reduces the irritation associated with sputum suction, fluctuation of ICP, cough response, and short-term complications, and improves the GOS score. These findings indicate that propofol should be used before ES to relieve the stress response of the patients undergoing the procedure.

### Propofol sedation before ES helps to stabilize intracranial pressure

ES is an effective method for keeping the artificial airway unobstructed in patients with severe neurologic diseases, and is, therefore, the most common procedure in the neurological ICU. However, ES can increase ICP by stimulating the airway mucosa, triggering cough reflex, elevating chest pressure, increasing blood flow into the brain, and decreasing venous return. The variations in the stimulation of the airway caused by the differences in suction methods, suction duration, negative pressure applied, and suction tube insertion depth, are reflected in ICP changes [4–6, 9–12]. Previous studies had demonstrated that ES was an important factor affecting ICP [6–8]. The results of the current work showed that the average peak value of ICP in the experimental group was 15.57 ± 12.31 mmHg, while that in the control group was 18.24 ± 8.99 mmHg. This finding indicates that propofol sedation before ES can effectively reduce the mean peak of ICP. This beneficial action of propofol depends on its ability to activate the GABA receptor chloride complex and decrease the stress response of the body caused by ES. Moreover, propofol can reduce cerebral blood flow, ICP, and CMRO2 [13–16].

### Propofol sedation ensures ES effect

Patients undergoing major neurosurgery procedures often experience consciousness disorders and reduced ability of the respiratory tract to perform self-cleaning. It is necessary to conduct timely suction of the sputum and clear respiratory secretion to avoid the obstruction of the artificial airway and pulmonary infection [1, 4, 5]. The sputum suction tube repeatedly stimulates the respiratory mucosa, resulting in varying degrees of choking and coughing in patients. In severe cases, it causes a decrease in blood oxygen saturation and an increase in ICP, producing discomfort [4–8]. Propofol is a short-term anesthesia drug, which is rapidly distributed in the entire organism within 40 s after intravenous injection. Intravenous injection of propofol before ES produces a sedative effect, inducing patients to enter the sleep state quickly. In addition, propofol can directly dilate bronchial smooth muscles, inhibit the throat reflex, and reduce the airway hyperresponsiveness during sputum suction. These properties of propofol suppress the stress response activated by ES and reduce the discomfort of patients [13, 14, 18].

### Propofol sedation before ES helps to improve the prognosis of patients undergoing major neurosurgery

In the present investigation, the concept of enhanced recovery after surgery (ERAS) were applied [16–18] to determine that an appropriate dose of sedatives was given before the ES according to the weight of the patients. The results showed that propofol did not cause the adverse reactions and complications. The evaluation of the GOS prognosis score sixth months after the operation revealed a high proportion of patients with 4–5 points on the GOS scale in the experimental group. These results indicated that the prognosis of patients treated with propofol was better. The collected data showed that propofol sedation before ES helped to improve the prognosis of patients undergoing major neurosurgery procedures by reducing the incidence of choking cough and spikes in ICP.

Some limitations of this study should be acknowledged. Firstly, only the patients admitted to the neurological ICU of the West China Hospital of Sichuan University were included. The subjects were mostly patients with cerebrovascular disease and severe brain injury. Secondly, we did not compare all the adverse effects of propofol including desaturation, recovery agitation, oversedation, agitation and so on [28], we only administered lidocaine to decrease the injection pain and evaluated the vital signs changes. Clinical multi-center trials involving a more extensive range of diseases, larger sample sizes and more comprehensive adverse effects of propofol are needed to support the conclusions.

## Conclusions

Sedation with a proper amount of propofol before ES could reduce the cough response caused by intense stimulation, reduce the patient’s painful experience, suppress the increase in ICP, and improve long-term prognosis. The administration of propofol was safe and does not affect the vital signs.

## Data Availability

The datasets used and/or analyzed during the current study available from the corresponding author on reasonable request.

## Abbreviations

ES: Endotracheal suctioning
ICP: Intracranial pressure
GOS: Glasgow Outcome Scale

## References

[1] Neurosurgery Branch of Chinese Medical Association (2016). China neurosurgery critical management cooperation group, expert consensus on airway management of Chinese neurosurgery critical patients. Chin Med J.

[2] Pedersen CM, Rosendahl-Nielsen M, Hjermind J, Egerod I (2009). Endotracheal suctioning of the adult intubated patient--what is the evidence?. Intensive Crit Care Nurs.

[3] Xue P, Gao J, Wenhua Z (2019). Research progress of sputum suction in artificial airway of patients with mechanical ventilation. Nurs Res.

[4] Neurosurgery Branch of Chinese Medical Association (2013). Experts consensus on neurosurgery critical management. Chin Med J.

[5] Kolb G, Brfker M (2009). State of the art in aspiration assessment and the idea of anew noninvasive predictive test for the risk of aspiration in stroke. J Nutri Heal Agi.

[6] Argent AC (2019). Endo tracheal Lidocaine installation, endotracheal suction, and pressure in patients with traumatic brain injury-assessing the impact. Pediatr Crit Care Med.

[7] Singh S, Chouhan RS, Bindra A, Radhakrishna N (2018). Comparison of effect of dexmedetomidine and lidocaine on intracranial and systemic hemodynamic response to chest physiotherapy and tracheal suctioning in patients with severe traumatic brain injury. J Anesth.

[8] Galbiai G, Paola C (2015). Effects of open and closed endotracheal suctioning on intracranial pressure and cerebral perfusion pressure in adult patients with severe brain injury: a literature review. J Neurosci Nurs.

[9] Favretto DO, de Campos Pereira Silveira RC, da Silva Canini SRM, Garbin LM, Martins FTM, Dalri MCB (2012). Endotracheal suction in intubated critically ill adult patients undergoing mechanical ventilation:a systematic review. Rev Lat Am Enfermage.

[10] Lai H-C, Pao S-I, Huang Y-S, Chan S-M, Lin B-F, Wu Z-F (2018). The relationship between postoperative pneumonia and endotracheal suctioning under general anesthesia in ophthalmic surgery: a retrospective study. Asian J Anesthesiol.

[11] Schults JA, Long DA, Mitchell ML, Cooke M, Gibbons K, Pearson K, Schibler A (2020). Adverse events and practice variability associated with paediatric endotracheal suction: An observational study. Aust Crit Care..

[12] Chegondi M, Francis T, Lin W-C, Naqvi S, Raszynski A, Totapally BR (2018). Effects of closed endotracheal suctioning on systemic and cerebral oxygenation and hemodynamics in children. Pediatr Crit Care Med.

[13] Severe Medicine Branch of Chinese Medical Association (2018). Chinese guidelines for analgesia and sedation in ICU for adults. Chinese Crit Emerg Med.

[14] Dapeng R, Wenjuan C, Wenqiang L, Wu T (2018). Effects of propofol combined with dexmedetomidine on circulatory system and sedation in patients with mechanical ventilation in intensive care unit. Trauma and Crit Illness Med.

[15] Abdemalik PA, Rakocevic G (2017). Propofol as a risk factor for ICU-acquired weakness in septic patients with acute respiratory failure. Can J Neurol Sci.

[16] Zhou Y, Jin X, Kang Y, Liang G, Liu T, Deng N (2014). Midazolam and propofol used alone or sequentially for long-term sedation in critically ill, mechanically ventilated patients: a prospective, randomized study. Crit Care.

[17] Kim S, Hahn S, Jang MJ, Choi Y, Hong H, Lee JH, Kim HS (2019). Evaluation of the safety of using propofol for paediatric procedural sedation: a systematic review and meta-analysis. Sci Rep.

[18] Zhu M, Yu K, Pan Y (2018). A randomized, double-blind, controlled study of postoperative sputum aspiration sedation by fiberoptic bronchoscopy in patients undergoing thoracic surgery. J Shanghai Jiaotong Univ (Medical Edition).

[19] Thomas M, Engelhardt T (2020). Is low-dose propofol sedation safe in unfasted patients?. Br J Anaesth..

[20] Chinese Neurosurgical Society (2020). Consensus of Chinese neurosurgery experts on critical care management (2020). Natl Med J China.

[21] Liangnan Z, Yang C (2017). Study on the depth of sputum suction in patients with tracheotomy in neurosurgery. Nurs Res.

[22] Maggiore SM, Lellouche F, Pignataro C, Girou E, Maitre B, Richard J-CM, Lemaire F, Brun-Buisson C, Brochard L (2013). Decreasing the adverse effects of endotracheal suctioning during mechanical ventilation by changing practice. Respir Care.

[23] Bin Z, Mao X, Jie F (2020). Analysis of factors influencing long-term prognosis of craniocerebral trauma. J Clin Neurosurgery.

[24] Takeuchi S, Takasato Y, Masaoka H, Nagatani K, Otani N, Wada K, Mori K (2015). Decompressive craniectomy for arteriovenous malformation-related intracerebral hemorrhage. J Clin Neurosci.

[25] Timmons SD, Bee T, Webb S, Diaz-Arrastia RR, Hesdorffer D (2011). Using the abbreviated injury severity and Glasgow coma scale scores to predict 2-week mortality after traumatic brain injury. J Trauma-Injury Infect Crit Care.

[26] Poon WS, Zhu XL, Ng SCP, Wong GKC (2004). Predicting one year clinical outcome in traumatic brain injury (TBI ) at the beginning of rehabilitation. Acta Neurochir Suppl.

[27] Dhandapani M, Dhandapani S, Agarwal M, Mahapatra AK (2014). Pressure ulcer in patients with severe traumatic brain injury: significant factors and association with neurological outcome. J Clin Nurs.

[28] Wakai A, Blackburn C, McCabe A, Reece E, O’Connor G, Glasheen J, Staunton P, Cronin J, Sampson C, McCoy SC, O’Sullivan R, Cummins F (2015). The use of propofol for procedural sedation in emergency departments. Cochrane Database Syst Rev.

